# Methylation of CALCA and CALCB in Pancreatic Ductal Adenocarcinoma

**DOI:** 10.1155/2021/2088345

**Published:** 2021-08-03

**Authors:** Feng Gao, Guozhong Liu, Jingwen Wang, Shirong Huang, Fadian Ding, Wei Lian, Xiaoting Lv, Yujia Guo, Xiangqun Fan, Sheng Zhang, Qicai Liu

**Affiliations:** ^1^Department of Pathology, 1st Affiliated Hospital, Fujian Medical University, Fuzhou 350005, China; ^2^Department of Hepatobiliary Surgery, 1st Affiliated Hospital, Fujian Medical University, Fuzhou 350005, China; ^3^Department of Neurology, Tiantai People's Hospital of Zhejiang Province, Tiantai 317200, China; ^4^Department of Laboratory Medicine, Fujian Medical University, 350004 Fuzhou, China; ^5^Department of Respiratory, 1st Affiliated Hospital, Fujian Medical University, Fuzhou 350004, China; ^6^Center for Reproductive Medicine, 1st Affiliated Hospital, Fujian Medical University, Fuzhou 350004, China; ^7^Center of Prenatal Screening, Fujian Provincial Maternity and Children's Hospital, Fujian Medical University, Fuzhou 350005, China

## Abstract

Calcitonin gene-related peptide (CGRP) plays a diverse and intricate role in chronic low-grade inflammation and is closely related to specific cancers. It includes two subtypes, CALCA (*α*CGRP) and CALCB (*β*CGRP), of which *α*CGRP expression accounts for more than 90%. Here, we show that methylation of CALCA and CALCB in pancreatic ductal adenocarcinoma was significantly higher than that in paracancer. Western blot and immunohistochemistry showed that CGRP, p-AKT, and p-CREB in the tumor tissues were lower than those in the paracarcinoma tissues. *In vivo*, the expressions of p-AKT and p-CREB in the pancreatic tissues of CALCA-KO rats were also lower than those of wild type. Methylation of CALCA and CALCB is increased in pancreatic adenocarcinoma, and under that condition, p-AKT and p-CREB levels were decreased.

## 1. Introduction

With the mortality approximately equal to the morbidity [1], pancreatic cancer has become one of the most fatal malignant tumors, among which pancreatic ductal carcinoma accounts for more than 90% [2]. However, the mechanism of occurrence and development of pancreatic cancer is still unclear. DNA methylation is an important epigenetic modification, which can regulate cell proliferation, apoptosis, gene expression, and stability, and is closely related to the tumor [3, 4].

Calcitonin gene-related peptide (CGRP) is a member of the calcitonin family of peptides, which can act as a growth or survival factor for several tumors, including endocrine-related tumors [5, 6]. The function of CGRP in stimulating angiogenesis and lymphangiogenesis may be one of the mechanisms [6]. There are two types that exist in CGRP: (1) *α*CGRP: the product of alternative splicing of the calcitonin gene (CT/CALCA) in neurons and whose expression accounts for more than 90% and is involved in regulating the function of various organs and (2) calcitonin gene-related peptide beta (CALCB), which has been discovered to form *β*CGRP, primarily expressed in the enteric sensory system, gut, and inner organs [5–7]. CGRP is one of the strongest vasoactive peptides found in vivo so far, which plays a role in relaxing and inhibiting vascular smooth muscle proliferation [6, 7]. At the same time, CGRP can stagnate the cell cycle in G0/G1 phase and thus participate in the regulation of tumor growth [8, 9].

The literature shows that CGRP plays a key role in the regulation of apoptosis and oxidative stress through the PI3K/Akt pathway [10]. Our previous work showed that abnormal CGRP can drive cell cycle disorder [11]. Therefore, we speculate that CGRP regulated oxidative stress injury, and cell cycle disorder plays an important role in the pathogenesis of pancreatic cancer. However, the epigenetics and expression of CGRP in pancreatic cancer are still unclear.

Therefore, the aims of this study were to investigate the methylation levels of CpG island in the CGRP promoter region of patients with pancreatic cancer by various methods to confirm the relationship between CGRP methylation, CGRP deficiency, and pancreatic cancer. *In vivo*, CGRP knockout rats (CALCA-KO) were also established to explore not only the relationship between CGRP deficiency and pancreatic cancer but also its possible pathogenesis.

## 2. Materials and Methods

### 2.1. Study Population

The data of sixty-three patients with pancreatic ductal adenocarcinoma hospitalized at the First Affiliated Hospital were enrolled (45 males and 18 females, male/female ratio≈5 : 2, range 43-72 years, mean age 57.5 years) between April 2015 and December 2019. In addition, fifteen healthy controls (10 males and 5 females, male/female ratio = 2 : 1, aged 35-60 years, median age 47.5 years) were selected from the physical examination center of our hospital during the same period. Patients were investigated for basic information, including the age of onset and family history. This research project was approved by the Ethics Committee of Fujian Medical University.

### 2.2. Pyrosequencing

DNA was extracted from peripheral blood, cancer tissues, and adjacent tissues of patients with pancreatic cancer using the Tiangen DNA Extraction Kit (Tiangen, Beijing, China). DNA was sent directly to Gene Tech (Shanghai) Co., Ltd. (Gene Tech, Shanghai, China) for pyrosequencing to detect the methylation level of CpG island in the CGRP promoter region.

### 2.3. DNA Bisulfite Conversion and Methylation Detection

The bisulfite conversion of DNA was carried out using Qiagen's EpiTect Fast DNA Bisulfite Kit. The initial sample dose was 1-2 mg, and the procedure was carried out according to the kit instructions. The 0.2 mL PCR tube was added with 20 *μ*L DNA, 85 *μ*L freshly prepared bisulfite conversion solution, and 35 *μ*L DNA protection solution in turn and then was mixed and put into the PCR instrument. The thermal cycle program was performed under the following conditions (step: temperature, time): Step 1: 95°C, 5 min; Step 2: 60°C, 10 min, a total of 2 cycles. Afterwards, conversion products were purified (Table 1).

### 2.4. Pathological Verification of CGRP-KO Rat Model

In order to investigate whether hypermethylation of CpG island in CGRP may promote the occurrence of pancreatic cancer, we constructed a CGRP knockout rat model to observe the changes in pancreatic tissue after CGRP-KO and to explore the possible mechanism. The model was constructed by GemPharmatech Co., Ltd. The rats were fed at room temperature of 22 ± 2°C and humidity of 40.00%-60.00%. Adequate rat food was given every day, drinking water was resteamed before being drunk, and the bedding material was changed at least twice a week to ensure that the bedding material was clean. For mating of rats, CGRP-KO (CGRP knockout rats) (> at 8 weeks) were cooped with WT (wild-type rats), with a male to female ratio of 1 : 1 or 2 : 1.

### 2.5. Immunohistochemistry

Immunohistochemistry (IHC) was performed to detect the expression of CALCA (rabbit antihuman polyclonal antibody, 1 : 100 dilution; A11804, ABclonal, CN), CALCB (rabbit antihuman polyclonal antibody, 1 : 3200 dilution; A5523, ABclonal, CN), AKT (rabbit antihuman polyclonal antibody, 1 : 3200 dilution; AB105, ABclonal, CN), p-AKT1-S473mAb (rabbit antihuman polyclonal antibody, 1 : 1600 dilution; AP0637, ABclonal, CN), CREB (rabbit antihuman polyclonal antibody, 1 : 1600 dilution; A10826, ABclonal, CN), and p-CREB-S133pAb (rabbit anti-human polyclonal antibody, 1 : 1600 dilution; AP0333, ABclonal, CN) proteins, according to the manufacturer's instructions. HRP-conjugated secondary antibody and DAB kit (Dako, Agilent Technologies, USA) were used to visualize antibody binding. Immunostaining reactivity was observed by using light microscopy (Olympus BX-53 with CCD DP73). The optical density value was analyzed with the Motic Med 6.0 analysis system.

### 2.6. Immunofluorescence

Immunohistochemistry (IHC) was performed to detect the expression of CALCA (rabbit antihuman polyclonal antibody, 1 : 50 dilution; A11804, ABclonal, CN), CALCB (rabbit antihuman polyclonal antibody, 1 : 200 dilution; A5523, ABclonal, CN), AKT (rabbit antihuman polyclonal antibody, 1 : 200 dilution; AB105, ABclonal, CN), p-AKT1-S473mAb (rabbit antihuman polyclonal antibody, 1 : 100 dilution; AP0637, ABclonal, CN), CREB (rabbit antihuman polyclonal antibody, 1 : 100 dilution; A10826, ABclonal, CN), and p-CREB-S133pAb (rabbit anti-human polyclonal antibody, 1 : 100 dilution; AP0333, ABclonal, CN) proteins, according to the manufacturer's instructions. The secondary antibody was rhodamine (TRI-TC)-conjugated goat anti-rabbit IgG or FITC-labeled goat anti-rabbit IgG. Nuclei were stained with DAPI solution.

### 2.7. Western Blot Analyses

Proteins from the pancreas of patients, CGRP-KO rats, and age-matched littermates were separated on 4 to 12% Tris-glycine gels and transferred to nitrocellulose membranes. Membranes were probed with antibodies directed against CALCA (rabbit anti-human polyclonal antibody, 1 : 100 dilution; A11804, ABclonal, CN), CALCB (rabbit anti-human polyclonal antibody, 1 : 3200 dilution; A5523, ABclonal, CN), AKT (rabbit anti-human polyclonal antibody, 1 : 3200 dilution; AB105, ABclonal, CN), p-AKT1-S473mAb (rabbit anti-human polyclonal antibody, 1 : 1600 dilution; AP0637, ABclonal, CN), CREB (rabbit anti-human polyclonal antibody, 1 : 1600 dilution; A10826, ABclonal, CN), and p-CREB-S133pAb (rabbit anti-human polyclonal antibody, 1 : 1600 dilution; AP0333, ABclonal, CN), and *β*-actin primary antibody (1 : 1000) was added and incubated overnight at 4°C. After washing with TBST for 10 min (3 times), the membrane was incubated with the corresponding secondary antibody (1 : 1000) and kept at room temperature for 2 h. After washing with TBST for 10 min (3 times), ECL developing solution (Beyotime, Shanghai, China, FFN02) was added, and development was carried out with a Bio-Rad gel imager to preserve the image.

### 2.8. Statistics

Statistical differences between groups were assessed by the nonparametric Mann-Whitney *U*-test for two groups and the Kruskal-Wallis test for more than two groups. Spearman's rank correlation coefficient estimated the degree of association between two variables. Significance was calculated at *P* < 0.05 by GraphPad Prism 5 (La Jolla, CA).

## 3. Results

### 3.1. CGRP Methylation Was Validated by Targeted Pyrosequencing Assays

**Figure 1:**
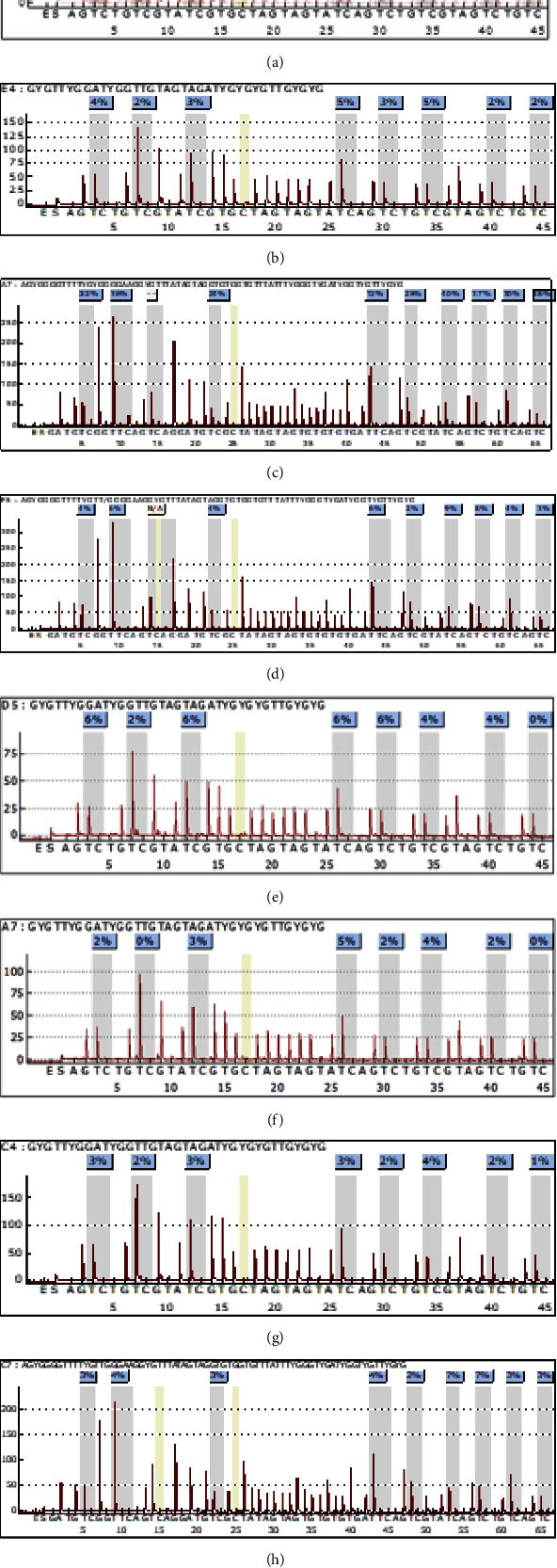
Pyrosequencing results of CALCA and CALCB: (a) CALCA pyrosequencing in pancreatic cancer tissue; (b) CALCA pyrosequencing in paracancer tissue; (c) CALCB pyrosequencing in pancreatic cancer tissue; (d) CALCB pyrosequencing in paracancer tissue; (e) CALCA pyrosequencing in peripheral blood of patients with pancreatic cancer; (f) CALCA pyrosequencing in peripheral blood of normal control; (g) CALCB pyrosequencing in peripheral blood of patients with pancreatic cancer; (h) CALCB pyrosequencing in peripheral blood of normal control.

Pyrosequencing was performed on the pancreatic ductal adenocarcinoma tissues, paracancer tissues, peripheral blood of the patients with pancreatic ductal adenocarcinoma, and healthy controls. It was found that the mean percentage of CpG island methylation in the CALCA promoter region in tissues of pancreatic ductal adenocarcinoma (13.14%) was significantly higher than that in paracancer tissues (3.00%, *P* = 0.0035) (Figures 1(a) and 1(b)). However, the mean percentage of CpG island methylation in CALCB was 13.57% in pancreatic cancer which is significantly higher than that in paracancer tissues (4.29%, *P* = 0.005) (Figures 1(c) and 1(d)). At the whole blood level, there was no statistically significant difference in the percentage of CpG island methylation in the CALCA (*P* = 0.1174) (Figures 1(e) and 1(f)) and the CALCB (*P* = 0.4481) (Figures 1(g) and 1(h)) promoter region between the patients with pancreatic cancer and normal controls.

### 3.2. Detection of CGRP Methylation by Bisulfite Sequencing PCR (BSP)

**Figure 2:**
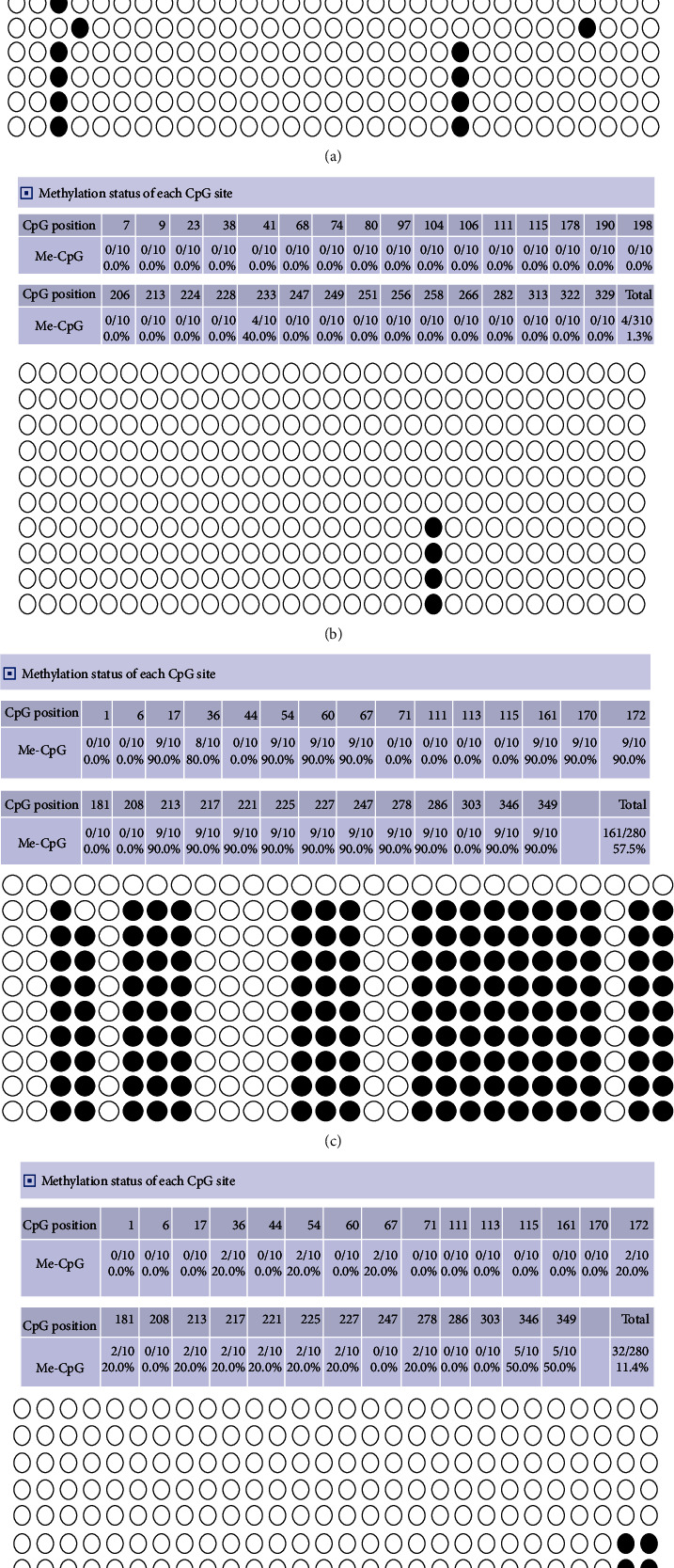
Detection of CGRP methylation by bisulfite sequencing PCR (BSP) in pancreatic cancer: (a) CALCA sequencing after pyrobisulfite modification in pancreatic cancer tissues; (b) CALCA sequencing after pyrobisulfite modification of the corresponding pancreatic cancer paratissue; (c) CALCB sequencing after pyrobisulfite modification in pancreatic cancer tissues; (d) CALCB sequencing after pyrobisulfite modification of the corresponding pancreatic cancer paratissue.

There were thirty CpG island sites in the promoter region of CALCA, with a methylation percentage of 4.2% in pancreatic cancer (Figure 2(a)) and 0.7% in paracancer tissues (Figure 2(b)) determined by BSP. Meanwhile, there were twenty-eight CpG island sites in the CALCB promoter region, with a methylation percentage of 57.5% in pancreatic cancer (Figure 2(c)) and 11.4% in paracancer tissues (Figure 2(d)) determined by BSP. The level of CGRP methylation in pancreatic ductal adenocarcinoma was higher than that in paracancer.

### 3.3. Detection of CGRP Methylation by Methylation-Specific PCR (MSP)

**Figure 3:**
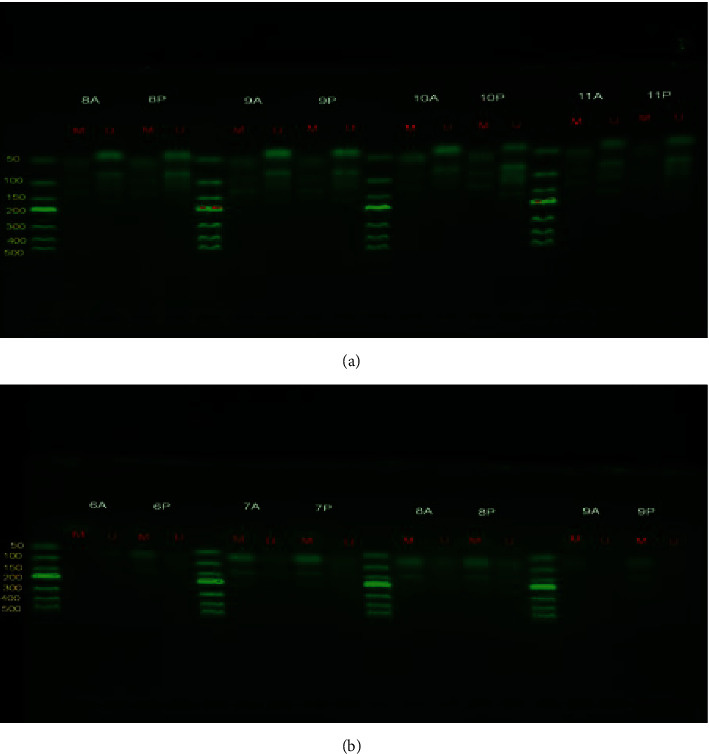
Detection of CGRP methylation by methylation-specific PCR (MSP): (a) CpG island methylation of CALCA was found by MSP electrophoresis in pancreatic cancer tissue. A: carcinoma; P: paracancer; M: methylation (target fragment: 132 bp); U: nonmethylation (target fragment: 158 bp). (b) CpG island methylation of CALCB was found by MSP electrophoresis in pancreatic cancer tissue. A: carcinoma; P: paracancer; M: methylation (target fragment: 143 bp); U: nonmethylation (target fragment: 105 bp). There is only one band of M in complete methylation, two bands of M and U in partial methylation, and only one band of U in nonmethylation.

CpG island methylation of CALCA was found in 85.71% (54/63) pancreatic ductal adenocarcinoma tissues. However, there was only 57.14% (36/63) found in paracancer tissues. Among the pancreatic ductal adenocarcinoma tissues, 39 cases presented complete methylation of CALCA (61.90%), and 15 cases presented partial methylation (23.81%). However, in paracancer tissues, the complete methylation of CALCA was observed in only two cases (3.17%), partial methylation in 34 paracancer (53.97%), and no methylation in 20 paracancer (31.75%) (Figure 3(a)).

For CALCB, there were 88.89% (56/63) patients showing methylation in the CpG island in pancreatic ductal adenocarcinoma, while only 46.03% (29/63) patients showed methylation in the paracancer tissue. In pancreatic ductal adenocarcinoma, 32 patients showed complete methylation of CALCB (50.79%), and 24 patients showed partial methylation (38.10%). In paracancer tissues, partial methylation of CALCB was observed in 29 paracancer (46.03%), and no methylation was observed in 31 paracancer (49.21%) (Figure 3(b)). The results of MSP indicated that the level of CGRP methylation in pancreatic ductal adenocarcinoma was higher than that in paracarcinoma.

### 3.4. Expression of CGRP in Pancreatic Cancer

**Figure 4:**
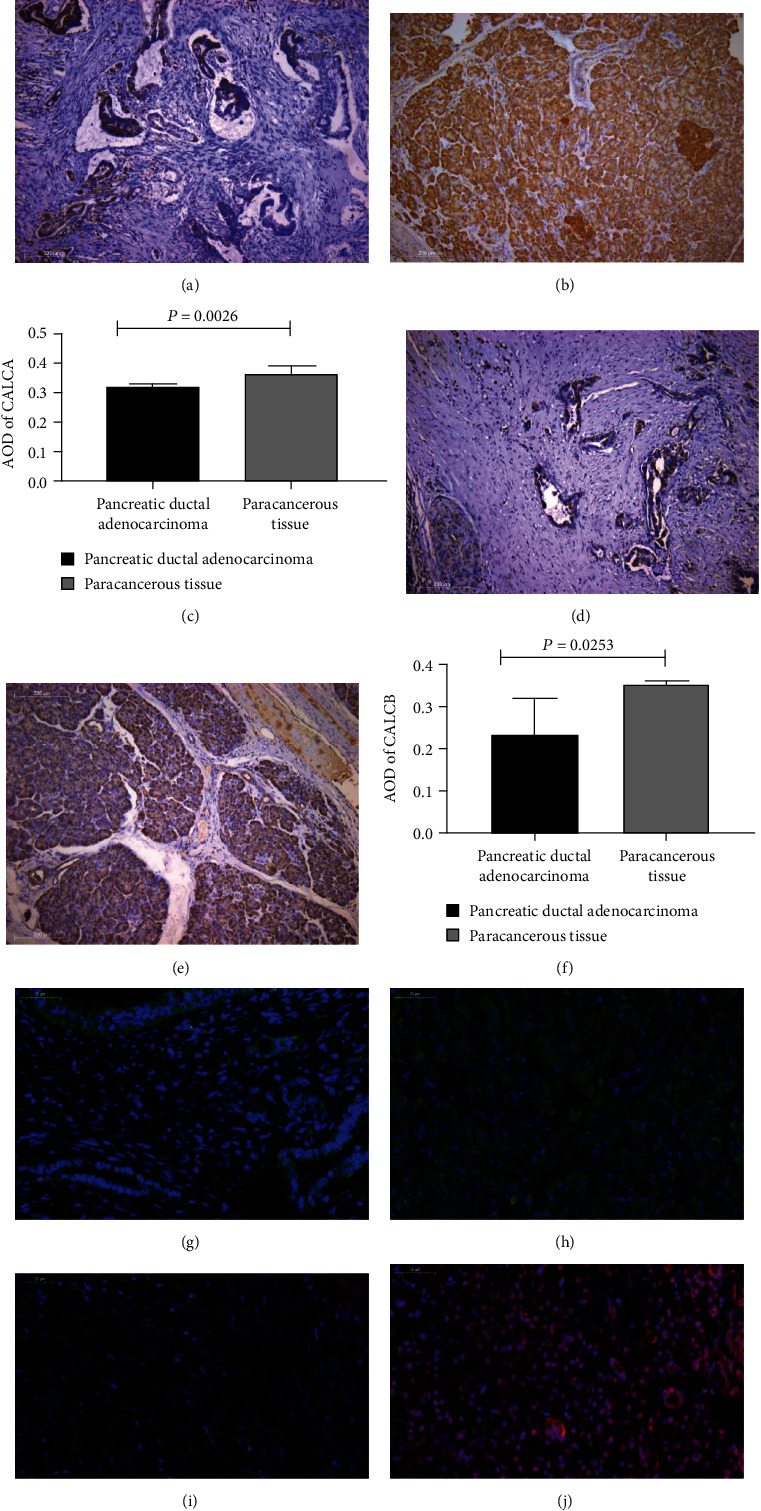
Expression of CGRP in pancreatic cancer and adjacent tissues: (a) expression of CALCA in pancreatic cancer tissues (IHC, ×100); (b) expression of CALCA in paracancer tissues (IHC, ×100); (c) differences of CALCA expression between pancreatic cancer tissues and adjacent tissues (IHC, ×100); (d) expression of CALCB in pancreatic cancer tissues (IHC, ×100); (e) expression of CALCB in paracancer tissues (IHC, ×100); (f) differences in CALCB expression between pancreatic cancer tissues and adjacent tissues (IHC, ×100); (g) expression of CALCA in pancreatic cancer tissues (immunofluorescence, ×400); (h) expression of CALCA in paratissue (immunofluorescence, ×400); (i) expression of CALCB in pancreatic cancer tissues (immunofluorescence, ×400); (j) expression of CALCB in paracancer tissues (immunofluorescence, ×400).

Both CALCA (*α*CGRP) and CALCB (*β*CGRP) showed lower expression in pancreatic ductal adenocarcinoma tissues than those in normal tissues far away from carcinoma (*P* < 0.05) (Figures 4(a)–4(f)). Consistently, immunofluorescence also showed that the two subtypes of CGRP were lower expressed in pancreatic ductal adenocarcinoma tissues than those in normal pancreatic tissues (Figures 4(g)–4(j)).

### 3.5. AKT-CREB Pathway Changes in the Case of CGRP Methylation Validated by Western Blot

**Figure 5:**
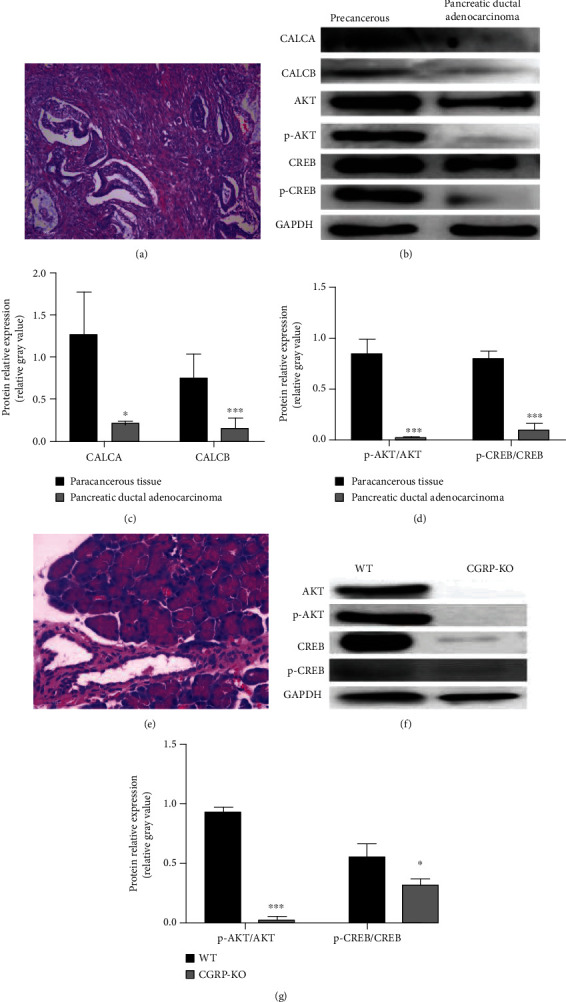
The possible mechanism of hypermethylation of CGRP promotes the development of pancreatic ductal adenocarcinoma: (a) histopathology of pancreatic ductal adenocarcinoma; (b) Western blot results of pancreatic ductal adenocarcinoma; (c, d) Western blot and grayscale analysis of pancreatic ductal adenocarcinoma; (e) H&E staining of pancreatic tissue from CGRP-KO rat; (f) Western blot results of pancreatic tissue from CGRP-KO rat; (g) Western blot and grayscale analysis of pancreatic tissue from CGRP-KO rat.

CALCA, CALCB, p-CREB/CREB, and p-AKT/AKT in pancreatic ductal adenocarcinoma tissues were downregulated compared with the corresponding paracancer tissues (*P* < 0.001) (Figures 5(a)–5(d)). The expression of p-AKT/AKT and p-CREB/CREB in the pancreatic tissue of CGRP-KO rats was downregulated compared with the wild type (*P* < 0.05) (Figures 5(e)–5(g)).

### 3.6. Immunohistochemical Staining to Verify AKT-CREB Pathways

**Figure 6:**
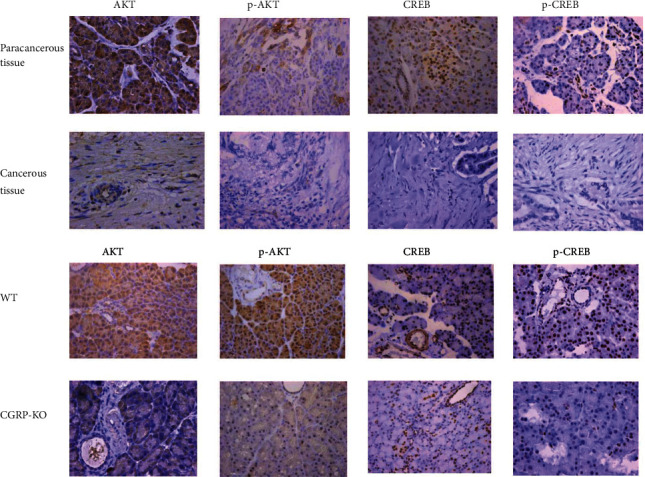
Immunohistochemical staining to verify AKT-CREB pathways affected by CGRP deficiency.

The results of immunohistochemistry showed that the expression of AKT (*P* = 0.0123), p-AKT (*P* = 0.0357), CREB (*P* = 0.0473), and p-CREB (*P* = 0.0256) in pancreatic ductal adenocarcinoma was lower than that of paracarcinoma (Figure 6).

*In vivo*, AKT (*P* = 0.0008), p-AKT (*P* = 0.0026), CREB (*P* = 0.0399), and p-CREB (*P* = 0.0256) in the pancreatic tissue of CGRP-KO rats were lower than those of the wild type (Figure 6).

### 3.7. The Relationship between CpG Island Methylation in CGRP Promoter Region and Pancreatic Cancer

**Figure 7:**
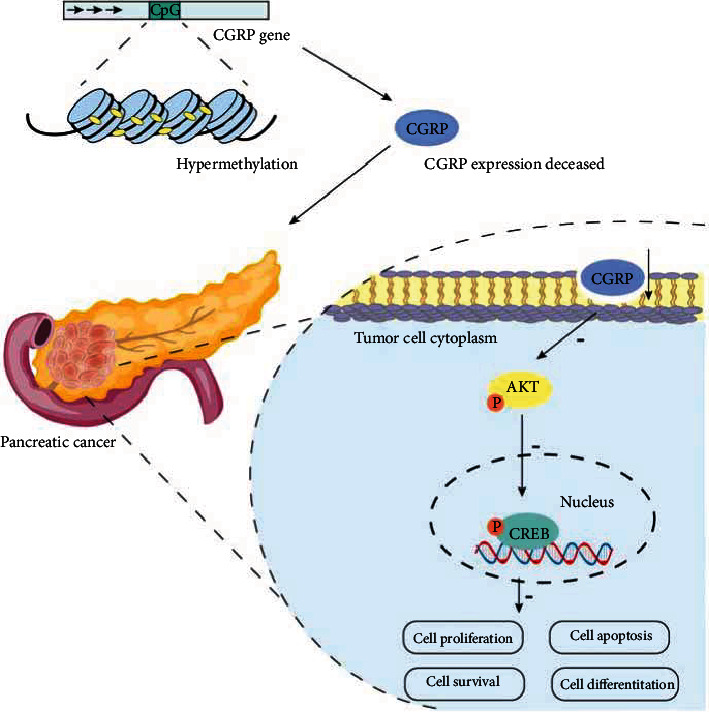
Relationship between CpG island methylation in CGRP and the occurrence and development of pancreatic cancer.

The hypermethylation of CpG island in the CGRP promoter region leads to low expression of CGRP, which affects the AKT-CREB pathway, thus promoting the development of pancreatic cancer (Figure 7).

## 4. Discussion

Pyrosequencing showed that methylation of CGRP in pancreatic ductal adenocarcinoma was significantly higher than that in paracancer. BSP and MSP also showed that the methylation level of CpG islands in the promoter region of CGRP in pancreatic cancer tissues was higher than that in paracancerous, indicating that CGRP hypermethylation plays an important role in the development of pancreatic cancer. However, the results of peripheral blood pyrosequencing demonstrated that there was no significant difference in CGRP methylation between pancreatic cancer patients and normal controls, indicating that CGRP methylation had tissue and organ specificity.

Immunohistochemical staining and immunofluorescence showed that the expression of CGRP in pancreatic cancer tissues was significantly reduced compared with normal pancreatic tissues. Therefore, we speculated that the hypermethylation of CGRP caused the low expression of CGRP and promoted the development of pancreatic cancer. In order to further explore the relationship between hypermethylation or low expression of CGRP and pancreatic cancer, we constructed CGRP-KO models to further explore the function of CGRP.

It was reported that CGRP has a wide range of biological activities, and lower expression of CGRP can promote tumor growth through its ability to promote angiogenesis [12, 13]. The best-known function of CGRP is its effect on the peripheral vasculature. Also, it has been known to modulate the neuromuscular junctions by inhibiting the expression of acetylcholinesterase, which is involved in inflammation within the airways, gastric secretions, and intestinal mobility [12, 13]. It may dampen the immune response primarily by modifying antigen presentation in a variety of antigen-presenting cells and stimulating naive T cells in the primary immune response [14–16]. Tumor development depends on the tumor vascular network to provide adequate oxygen and nutrients, and tumor angiogenesis depends on highly complex growth factor signaling, endothelial cell (EC) proliferation, and other functions [14]. Moreover, CGRP could block the cell cycle from G0/G1 phase to S phase, which may be an important reason for its involvement in tumorigenesis [15, 16]. However, the mechanism by which CGRP deficiency causes pancreatic cancer is not clear.

Protein kinase B (PKB/AKT) plays an important role in carcinogenesis and cell growth control [10, 17], and CGRP activates AKT, which phosphorylates CREB at Ser133 and regulates CREB-mediated gene transcription [18, 19]. The AKT-CREB signaling pathway is involved in cell proliferation, apoptosis, differentiation, and survival, and it affects the occurrence and development of tumors [20, 21]. In this study, Western blot results of pancreatic cancer tissues showed that the expression levels of p-AKT/AKT and p-CREB/CREB were both lower than paracancer, and the results were verified by the CGRP knockout model. To further confirm it, we observed downregulation of both AKT and CREB proteins in pancreatic cancer tissue. The deregulated protein expression of the AKT-CREB pathway was also found in CGRP-KO rats.

## Figures and Tables

**Table 1:** Primer sequences used to amplify CGRP.

	Target gene (promoter region)	CALCA (NC_000011.10 (14966668…14972361, complement))
MSP	Forward	5′-TTTTAGGTTTTGGAAGTATGAGGGTGATG-3′
	Reverse	5′-TTCCCACCACTATAAATCA-3′
	Annealing temperature	53°C

USP	Forward	5′-GTTTTGGAAGTATGAGGGTGACG-3′
	Reverse	5′-TTCCCGCCGCTATAAATCG-3′
	Annealing temperature	53°C
	Target gene (promoter region)	CALCB (NC_000011.10 (15073593…15078637))

MSP	Forward	5′-TTTTTAGAAAAGATGGATAGGTCGA-3′
	Reverse	5′-ACCAACACTCACTAAAACAAATACG-3′
	Annealing temperature	45°C

USP	Forward	5′-TTTTTAGAAAAGATGGATAGGTTGA-3′
	Reverse	5′-CCAACACTCACTAAAACAAATACAC-3′
	Annealing temperature	45°C

## Data Availability

The data used to support the findings of this study are currently under embargo while the research findings are commercialized. Requests for data, 12 months after publication of this article, will be considered by the corresponding authors.
